# 93 Inhalation Injury: Which Providers Can Assess the Need for Intubation?

**DOI:** 10.1093/jbcr/irad045.066

**Published:** 2023-05-15

**Authors:** Laura Adams, Louis Perkins, Jarrett Santorelli, Jeanne Lee, Alan Smith, Jay Doucet

**Affiliations:** UCSD, San Diego, California; University of California San Diego, San Diego, California; University of California San Diego, San Diego, California; UC San Diego Health, San Diego, California; UC San Diego, San Diego, California; UC San Diego, San Diego, California; UCSD, San Diego, California; University of California San Diego, San Diego, California; University of California San Diego, San Diego, California; UC San Diego Health, San Diego, California; UC San Diego, San Diego, California; UC San Diego, San Diego, California; UCSD, San Diego, California; University of California San Diego, San Diego, California; University of California San Diego, San Diego, California; UC San Diego Health, San Diego, California; UC San Diego, San Diego, California; UC San Diego, San Diego, California; UCSD, San Diego, California; University of California San Diego, San Diego, California; University of California San Diego, San Diego, California; UC San Diego Health, San Diego, California; UC San Diego, San Diego, California; UC San Diego, San Diego, California; UCSD, San Diego, California; University of California San Diego, San Diego, California; University of California San Diego, San Diego, California; UC San Diego Health, San Diego, California; UC San Diego, San Diego, California; UC San Diego, San Diego, California; UCSD, San Diego, California; University of California San Diego, San Diego, California; University of California San Diego, San Diego, California; UC San Diego Health, San Diego, California; UC San Diego, San Diego, California; UC San Diego, San Diego, California

## Abstract

**Introduction:**

Previous studies have suggested many burn patients undergo unnecessary intubation due to concern for inhalation injury. There is a lack of clear consensus guidelines for intubation and thus there is significant variation in practice pattern. In this study we hypothesized intubation rates would differ between Acute Care Surgeons with specialized burn training and those without.

**Methods:**

A retrospective analysis of 388 patients admitted to an ABA verified level 1 burn center who presented emergently following burn injury from 7/2015 to 12/2021 was performed. Patients excluded include polytrauma patients, isolated friction burns, and patients intubated prior to hospital arrival. All providers performing initial evaluation are Acute Care Surgeons, with a subset having additional specialized fellowship training and/or extensive burn experience. Our primary outcome was intubation rates between burn and non-burn trained providers. The Chi-Square and Mann-Whitney U statistical tests were used to compare categorical and continuous variables, respectively.

**Results:**

There were 465 major burn patients admitted through the trauma bay and screened during the study period with 388 patients meeting inclusion criteria. The population was 72% male, with mean age of 44 years +- 21, TBSA of 12%, and COHgb level of 2.9%. In total, 73 (19%) of patients underwent intubation. The patients in the two provider groups did not differ in terms of age, gender, race, TBSA, COHgb level, or airway exam findings. 240 (62%) patients were evaluated by a burn provider and 148 (38%) were evaluated by a non-burn provider. There was no significant difference in the rate of emergent intubation of patients treated by burn vs non-burn trained providers (RR 0.71, CI 0.47 - 1.07). Of the patients who were intubated, there was no significant difference between burn and non-burn providers in the diagnosis of inhalation injury on bronchoscopy (RR 1.3, CI 0.91-1.85). In both groups, objective evidence of inhalation injury was found in only 68% of patients undergoing routine bronchoscopy following emergent intubation.

**Conclusions:**

There was no significant difference in intubation rates between burn and non-burn trained acute care surgeons. This demonstrates that following standard ATLS and ABLS principles provides appropriate training in the evaluation, diagnosis, and treatment of inhalation injury. Interestingly, 32% of all patients intubated did not have evidence of inhalational injury on bronchoscopy following intubation. This study suggests that further research is needed to determine objective criteria for intubation guidelines when inhalation injury is suspected in the acute burn patient population.

**Applicability of Research to Practice:**

Applicable

